# Supplementary material for: Distributions of p-values smaller than .05 in psychology: what is going on?
Source: PeerJ. 2016 Apr 11;4:e1935. doi: 10.7717/peerj.1935 (PMC4830257; doi:10.7717/peerj.1935)
Supplement: File S1 [file peerj-04-1935-s001.pdf]

Supplemental file to Hartgerink, van Aert, Nuijten, Wicherts, and van Assen

March 22, 2016

Modeling p-value distributions

For both measures from the previous section the expected p-value distribution needs to be derived and compared to the observed p-value distribution. The observed p-value distribution of the psychology data is based on all exactly reported statistics with test statistics t, r, and $F(1, df_2)$, because these readily provide the same effect measure. We used the Fisher transformed correlation, ρ_F, as effect size measure. The distribution of the Fisher transformed correlation is approximated well by the normal distribution, with Fisher transformation

$$\rho_F = \frac{1}{2} ln(\frac{1+r}{1-r}) \tag{1}$$

and standard error $\frac{1}{\sqrt{N-3}}$ or $\frac{1}{\sqrt{df_2-1}}$. $F(1, df_2)$ and t values can be transformed to correlations using

$$r = \frac{\frac{F \times df_1}{df_2}}{\frac{F \times df_1}{df_2} + 1} \tag{2}$$

where $F = t^2$.

The expected p-value distribution was estimated under the assumption that the true effect size, Fisher transformed correlation ρ_F, is normally distributed with mean effect δ_{ρ_F} and standard deviation τ_{ρ_F}. The two parameters were estimated by minimizing the χ^2-statistic

$$\chi^2_{j-1} = \sum_{j=1}^{J} \frac{(rf^o_j - rf^e_j)^2}{rf^e_j} \tag{3}$$

with rf^o and rf^e being the relative frequency of observed and expected p-values in interval j, respectively. Minimization was done with the `optim()` function in R, where $\hat{\tau}$ was truncated to be positive. Interval j is defined as $(I_{j-1}, I_j) = ((j-1)x, jx))$, with width $x = .00025$ whenever only the significant p-values lower than .00125 were modeled (resulting in 5 intervals); .00125 when modeling all significant p-values (i.e., $p \leq .05$, 40 intervals); .025 when modeling all p-values (also 40 intervals). The relative frequencies are conditional probabilities. For instance, rf^o_2 is the proportion of observed p-values in interval $(I_1 = .00025, I_2 = .0005)$ whenever p-values lower than .00125 are examined. Expected relative

frequency rf_j^e is computed as

$$rf_j^e = \frac{\sum_{k=1}^{K} P(I_{j-1} \leq p_k \leq I_j | N_k; \hat{\rho}_F; \hat{\tau}_{\rho_F})}{\sum_{k=1}^{K} P(p_k \leq I_J | N_k; \hat{\rho}_F; \hat{\tau}_{\rho_F})} \tag{4}$$

with the summation over all K significant test statistics. P corresponds to the probability that a p-value of study k (i.e., p_k) is in a certain interval, which depends on the study sample size N_k and the two estimated parameters of the effect size distribution (i.e., $\hat{\rho}_F, \hat{\tau}_{\rho_F}$).
